# (2*E*)-3-(3,4-Dimeth­oxy­phen­yl)-1-(4-hy­droxy­phen­yl)prop-2-en-1-one

**DOI:** 10.1107/S1600536811007781

**Published:** 2011-03-09

**Authors:** Jerry P. Jasinski, Ray J. Butcher, V. Musthafa Khaleel, B. K. Sarojini, B. Narayana

**Affiliations:** a Department of Chemistry, Keene State College, 229 Main Street, Keene, NH 03435-2001, USA; b Department of Chemistry, Howard University, 525 College Street NW, Washington, DC 20059, USA; c Department of Chemistry, P.A. College of Engineering, Mangalore, 574 153, India; d Department of Studies in Chemistry, Mangalore University, Mangalagangotri, Mysore 574 199, India

## Abstract

In the title compound, C_17_H_16_O_4_, the dihedral angle between the mean planes of the hy­droxy­phenyl and dimeth­oxy­phenyl rings is 19.34 (7)°. The mean plane of the prop-2-en-1-one group, the active site in this mol­ecule, makes angles of 7.40 (8) and 13.25 (8)°, respectively, with the hy­droxy­phenyl and dimeth­oxy­phenyl rings. The crystal packing is stabilized by O—H⋯O hydrogen bonds, weak inter­molecular C—H⋯O and π–π stacking inter­actions [centroid–centroid distance = 3.7386 (9) Å].

## Related literature

For related chalcone structures, see: Butcher *et al.* (2006 ▶); Cao *et al.* (2005 ▶); Harrison *et al.* (2007 ▶); Jasinski *et al.* (2010 ▶, 2011*a* ▶,*b* ▶); Ngaini *et al.* (2009 ▶); Radha Krishna *et al.* (2005 ▶); Sharma *et al.* (1997 ▶); Wu *et al.* (2005 ▶)
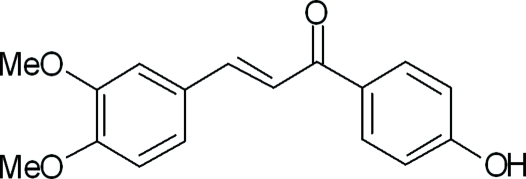


## Experimental

### 

#### Crystal data

C_17_H_16_O_4_
*M* *_r_* = 284.30
Orthorhombic, 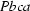

*a* = 15.1435 (1) Å
*b* = 8.4364 (1) Å
*c* = 22.9454 (2) Å
*V* = 2931.43 (5) Å^3^
*Z* = 8
Cu *K*α radiation
μ = 0.75 mm^−1^
*T* = 295 K
0.57 × 0.28 × 0.19 mm

#### Data collection

Oxford Diffraction Gemini R diffractometer
Absorption correction: multi-scan (*CrysAlis RED*; Oxford Diffraction, 2007 ▶) *T*_min_ = 0.606, *T*_max_ = 1.000
9187 measured reflections
3022 independent reflections
2545 reflections with *I* > 2σ(*I*)
*R*_int_ = 0.019

#### Refinement

*R*[*F* ^2^ > 2σ(*F* ^2^)] = 0.044
*wR*(*F* ^2^) = 0.132
*S* = 1.05
3022 reflections
193 parameters
H-atom parameters constrained
Δρ_max_ = 0.18 e Å^−3^
Δρ_min_ = −0.19 e Å^−3^

### 

Data collection: *CrysAlis PRO* (Oxford Diffraction, 2007 ▶); cell refinement: *CrysAlis PRO*; data reduction: *CrysAlis RED* (Oxford Diffraction, 2007 ▶); program(s) used to solve structure: *SHELXS97* (Sheldrick, 2008 ▶); program(s) used to refine structure: *SHELXL97* (Sheldrick, 2008 ▶); molecular graphics: *SHELXTL* (Sheldrick, 2008 ▶); software used to prepare material for publication: *SHELXTL*.

## Supplementary Material

Crystal structure: contains datablocks global, I. DOI: 10.1107/S1600536811007781/tk2725sup1.cif

Structure factors: contains datablocks I. DOI: 10.1107/S1600536811007781/tk2725Isup2.hkl

Additional supplementary materials:  crystallographic information; 3D view; checkCIF report

## Figures and Tables

**Table 1:** Hydrogen-bond geometry (Å, °)

*D*—H⋯*A*	*D*—H	H⋯*A*	*D*⋯*A*	*D*—H⋯*A*
O1—H1*A*⋯O2^i^	0.82	1.90	2.7154 (15)	176
C6—H6*A*⋯O4^ii^	0.93	2.46	3.2803 (16)	147

Symmetry codes: (i) 
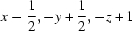
; (ii) 
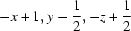
.

**Fig. 1.**
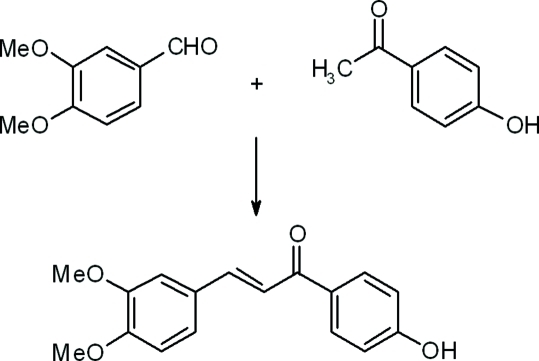
Reaction scheme for (I).

**Fig. 2.**
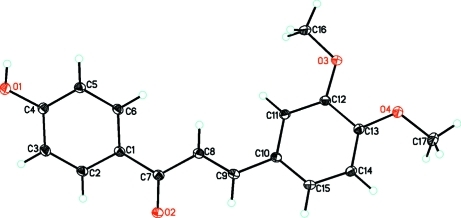
Molecular structure of the title compound showing the atom labeling scheme and 50% probability displacement ellipsoids.

**Fig. 3.**
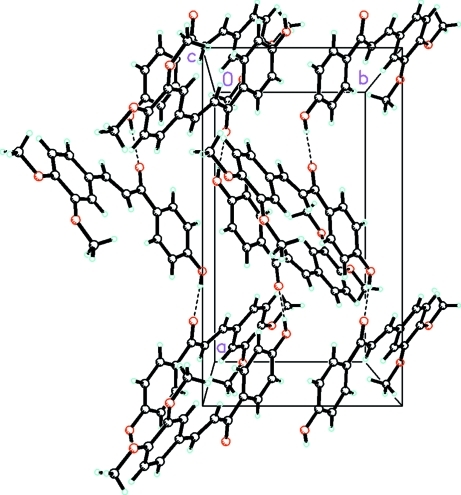
Packing diagram of the title compound viewed down the c axis. Dashed lines indicate O—H···O hydrogen bonds.

## supplementary crystallographic information

### Comment

In continuation to our studies on crystal structures of chalcones (Jasinski *et al.*, 2010, 2011*a*, 2011*b*), we report here the synthesis, Fig. 1, and crystal structure of a new chalcone, (I), Fig. 2. The dihedral angle between the mean planes of the hydroxyphenyl and dimethoxyphenyl rings is 19.34 (7)\ % (Fig. 2). The mean plane of the prop-2-en-1-one group, the active site in this molecule, makes angles of 7.40 (8) ° and 13.25 (8) °, respectively, with the hydroxyphenyl and dimethoxyphenyl rings. Bond lengths and angles are normal and correspond to those observed in related compounds (Butcher *et al.*, 2006; Cao *et al.*, 2005; Harrison *et al.*, 2007; Jasinski *et al.*, 2010, 2011*a*, 2011*b*; Ngaini *et al.*, 2009; Radha Krishna *et al.*, 2005; Sharma *et al.*, 1997; Wu *et al.*, 2005). Crystal packing is stabilized by O—H···O hydrogen bonds, weak C—H···O contacts (Table 2) and π—π stacking interactions (Table 3); see Fig. 3.

### Experimental

4-Hydroxyacetophenone (1.36 g, 0.01 mol) was mixed with 3,4-dimethoxybenzaldehyde (1.66 g, 0.01 mol) and dissolved in ethanol (40 ml), Fig.1. To this solution, 5 ml of KOH (50%) was added at 278 K. The reaction mixture stirred overnight at room temperature and poured on to crushed ice. The pH of this mixture was adjusted to 3–4 with 2 *M* HCl aqueous solution. The resulting crude solid was filtered, washed successively with dilute HCl solution and distilled water, and finally recrystallized from ethyl alcohol (95%) to give the pure chalcone. Crystals suitable for X-ray diffraction studies were grown by the slow evaporation of the solution of the compound in ethyl alcohol:DMF (4:1) (*M*.pt.: 438–442 K). Composition: Found (Calculated) for C_17_H_16_O_4_, C 75.22 (75.28); H: 5.95 (5.92) %.

### Refinement

The hydroxyl hydrogen was located by a Fourier map, fixed at 0.82 Å and refined using the riding model. All of the remaining H atoms were placed in their calculated positions and then refined using the riding model with C—H = 0.93 Å (CH) and 0.96 Å (CH_3_). Isotropic displacement parameters for these atoms were set to 1.19–1.20 (CH), 1.50 (CH_3_) or 1.50 (OH) times *U*_eq_ of the parent atom.

### Crystal data

**Table:**

C_17_H_16_O_4_	*F*(000) = 1200
*M**_r_* = 284.30	*D*_x_ = 1.288 Mg m^−^^3^
Orthorhombic, *P**b**c**a*	Cu *K*α radiation, λ = 1.54184 Å
Hall symbol: -P 2ac 2ab	Cell parameters from 5957 reflections
*a* = 15.1435 (1) Å	θ = 4.8–77.2°
*b* = 8.4364 (1) Å	µ = 0.75 mm^−^^1^
*c* = 22.9454 (2) Å	*T* = 295 K
*V* = 2931.43 (5) Å^3^	Thick needle, pale yellow
*Z* = 8	0.57 × 0.28 × 0.19 mm

### Data collection

**Table:**

Oxford Diffraction Gemini R diffractometer	3022 independent reflections
Radiation source: fine-focus sealed tube	2545 reflections with *I* > 2σ(*I*)
graphite	*R*_int_ = 0.019
Detector resolution: 10.5081 pixels mm^-1^	θ_max_ = 77.4°, θ_min_ = 4.8°
φ and ω scans	*h* = −19→16
Absorption correction: multi-scan (*CrysAlis RED*; Oxford Diffraction, 2007)	*k* = −10→8
*T*_min_ = 0.606, *T*_max_ = 1.000	*l* = −29→27
9187 measured reflections	

### Refinement

**Table:**

Refinement on *F*^2^	Primary atom site location: structure-invariant direct methods
Least-squares matrix: full	Secondary atom site location: difference Fourier map
*R*[*F*^2^ > 2σ(*F*^2^)] = 0.044	Hydrogen site location: inferred from neighbouring sites
*wR*(*F*^2^) = 0.132	H-atom parameters constrained
*S* = 1.05	*w* = 1/[σ^2^(*F*_o_^2^) + (0.0854*P*)^2^ + 0.2601*P*] where *P* = (*F*_o_^2^ + 2*F*_c_^2^)/3
3022 reflections	(Δ/σ)_max_ < 0.001
193 parameters	Δρ_max_ = 0.18 e Å^−^^3^
0 restraints	Δρ_min_ = −0.19 e Å^−^^3^

### Special details

**Table:**

Geometry. All e.s.d.'s (except the e.s.d. in the dihedral angle between two l.s. planes) are estimated using the full covariance matrix. The cell e.s.d.'s are taken into account individually in the estimation of e.s.d.'s in distances, angles and torsion angles; correlations between e.s.d.'s in cell parameters are only used when they are defined by crystal symmetry. An approximate (isotropic) treatment of cell e.s.d.'s is used for estimating e.s.d.'s involving l.s. planes.
Refinement. Refinement of *F*^2^ against ALL reflections. The weighted *R*-factor *wR* and goodness of fit *S* are based on *F*^2^, conventional *R*-factors *R* are based on *F*, with *F* set to zero for negative *F*^2^. The threshold expression of *F*^2^ > σ(*F*^2^) is used only for calculating *R*-factors(gt) *etc*. and is not relevant to the choice of reflections for refinement. *R*-factors based on *F*^2^ are statistically about twice as large as those based on *F*, and *R*- factors based on ALL data will be even larger.

### Fractional atomic coordinates and isotropic or equivalent isotropic displacement parameters (Å^2^)

**Table:**

	*x*	*y*	*z*	*U*_iso_*/*U*_eq_	
O1	0.35344 (7)	0.04833 (15)	0.56975 (5)	0.0633 (3)	
H1A	0.3054	0.0598	0.5538	0.095*	
O2	0.69247 (6)	0.40373 (15)	0.47932 (5)	0.0632 (3)	
O3	0.52144 (7)	0.68821 (14)	0.18477 (4)	0.0623 (3)	
O4	0.64891 (8)	0.87474 (16)	0.15770 (5)	0.0709 (4)	
C1	0.54831 (8)	0.30167 (16)	0.48339 (5)	0.0446 (3)	
C2	0.56558 (9)	0.21781 (19)	0.53484 (6)	0.0531 (3)	
H2A	0.6224	0.2190	0.5503	0.064*	
C3	0.50099 (9)	0.1343 (2)	0.56289 (6)	0.0565 (4)	
H3A	0.5142	0.0786	0.5968	0.068*	
C4	0.41531 (9)	0.13271 (17)	0.54069 (6)	0.0488 (3)	
C5	0.39652 (9)	0.21501 (18)	0.48989 (6)	0.0514 (3)	
H5A	0.3395	0.2144	0.4749	0.062*	
C6	0.46232 (9)	0.29769 (17)	0.46166 (6)	0.0486 (3)	
H6A	0.4491	0.3519	0.4275	0.058*	
C7	0.61991 (8)	0.39056 (17)	0.45507 (6)	0.0467 (3)	
C8	0.60426 (8)	0.46360 (16)	0.39746 (5)	0.0468 (3)	
H8A	0.5557	0.4317	0.3756	0.056*	
C9	0.65801 (8)	0.57388 (16)	0.37626 (6)	0.0470 (3)	
H9A	0.7045	0.6039	0.4004	0.056*	
C10	0.65336 (8)	0.65384 (15)	0.31995 (5)	0.0432 (3)	
C11	0.58497 (8)	0.62733 (15)	0.27941 (5)	0.0444 (3)	
H11A	0.5393	0.5580	0.2886	0.053*	
C12	0.58516 (8)	0.70317 (16)	0.22628 (5)	0.0457 (3)	
C13	0.65482 (9)	0.80698 (17)	0.21138 (6)	0.0481 (3)	
C14	0.72135 (9)	0.83476 (17)	0.25115 (6)	0.0517 (3)	
H14A	0.7671	0.9040	0.2420	0.062*	
C15	0.71971 (9)	0.75892 (18)	0.30489 (6)	0.0506 (3)	
H15A	0.7645	0.7794	0.3315	0.061*	
C16	0.44645 (10)	0.5955 (2)	0.19866 (7)	0.0639 (4)	
H16A	0.4646	0.4889	0.2071	0.096*	
H16B	0.4065	0.5949	0.1662	0.096*	
H16C	0.4174	0.6397	0.2321	0.096*	
C17	0.71898 (15)	0.9741 (3)	0.13898 (8)	0.0870 (6)	
H17A	0.7210	1.0672	0.1630	0.130*	
H17B	0.7095	1.0044	0.0991	0.130*	
H17C	0.7739	0.9179	0.1421	0.130*	

### Atomic displacement parameters (Å^2^)

**Table:**

	*U*^11^	*U*^22^	*U*^33^	*U*^12^	*U*^13^	*U*^23^
O1	0.0503 (5)	0.0816 (8)	0.0582 (6)	0.0019 (5)	0.0053 (4)	0.0155 (5)
O2	0.0424 (5)	0.0845 (8)	0.0628 (6)	−0.0018 (5)	−0.0107 (4)	0.0177 (6)
O3	0.0555 (6)	0.0791 (7)	0.0522 (5)	−0.0210 (5)	−0.0170 (4)	0.0141 (5)
O4	0.0766 (7)	0.0823 (8)	0.0539 (6)	−0.0329 (6)	−0.0121 (5)	0.0175 (6)
C1	0.0423 (6)	0.0489 (7)	0.0426 (6)	0.0079 (5)	−0.0023 (5)	−0.0020 (5)
C2	0.0424 (6)	0.0675 (9)	0.0495 (7)	0.0072 (6)	−0.0075 (5)	0.0054 (6)
C3	0.0520 (7)	0.0708 (9)	0.0469 (7)	0.0090 (7)	−0.0031 (5)	0.0115 (7)
C4	0.0457 (7)	0.0551 (8)	0.0455 (6)	0.0066 (6)	0.0049 (5)	−0.0012 (6)
C5	0.0405 (6)	0.0639 (8)	0.0496 (7)	0.0051 (6)	−0.0033 (5)	0.0023 (6)
C6	0.0446 (6)	0.0585 (8)	0.0428 (6)	0.0065 (5)	−0.0040 (5)	0.0048 (5)
C7	0.0414 (6)	0.0523 (7)	0.0464 (6)	0.0083 (5)	−0.0031 (5)	−0.0011 (5)
C8	0.0423 (6)	0.0549 (7)	0.0431 (6)	0.0039 (5)	−0.0028 (5)	−0.0027 (5)
C9	0.0441 (6)	0.0493 (7)	0.0476 (6)	0.0051 (5)	−0.0080 (5)	−0.0035 (5)
C10	0.0408 (6)	0.0442 (6)	0.0447 (6)	0.0039 (5)	−0.0034 (5)	−0.0034 (5)
C11	0.0387 (6)	0.0466 (7)	0.0479 (6)	−0.0022 (5)	−0.0028 (5)	−0.0003 (5)
C12	0.0415 (6)	0.0504 (7)	0.0451 (6)	−0.0033 (5)	−0.0053 (5)	−0.0026 (5)
C13	0.0493 (7)	0.0488 (7)	0.0463 (6)	−0.0054 (5)	−0.0011 (5)	−0.0003 (5)
C14	0.0462 (7)	0.0537 (7)	0.0553 (7)	−0.0111 (6)	−0.0019 (5)	−0.0017 (6)
C15	0.0428 (6)	0.0558 (8)	0.0533 (7)	−0.0036 (6)	−0.0105 (5)	−0.0049 (6)
C16	0.0474 (7)	0.0847 (11)	0.0596 (8)	−0.0175 (7)	−0.0120 (6)	0.0011 (8)
C17	0.0952 (14)	0.0961 (14)	0.0697 (10)	−0.0435 (12)	−0.0063 (9)	0.0232 (10)

### Geometric parameters (Å, °)

**Table:**

O1—C4	1.3524 (17)	C8—C9	1.328 (2)
O1—H1A	0.8200	C8—H8A	0.9300
O2—C7	1.2368 (16)	C9—C10	1.4592 (18)
O3—C12	1.3617 (15)	C9—H9A	0.9300
O3—C16	1.4153 (17)	C10—C15	1.3837 (19)
O4—C13	1.3608 (17)	C10—C11	1.4100 (16)
O4—C17	1.419 (2)	C11—C12	1.3768 (18)
C1—C6	1.3947 (18)	C11—H11A	0.9300
C1—C2	1.4009 (18)	C12—C13	1.4131 (18)
C1—C7	1.4698 (19)	C13—C14	1.3794 (19)
C2—C3	1.367 (2)	C14—C15	1.389 (2)
C2—H2A	0.9300	C14—H14A	0.9300
C3—C4	1.3940 (19)	C15—H15A	0.9300
C3—H3A	0.9300	C16—H16A	0.9600
C4—C5	1.3863 (19)	C16—H16B	0.9600
C5—C6	1.378 (2)	C16—H16C	0.9600
C5—H5A	0.9300	C17—H17A	0.9600
C6—H6A	0.9300	C17—H17B	0.9600
C7—C8	1.4775 (17)	C17—H17C	0.9600
			
C4—O1—H1A	109.5	C15—C10—C11	118.05 (12)
C12—O3—C16	117.54 (11)	C15—C10—C9	118.83 (11)
C13—O4—C17	118.23 (12)	C11—C10—C9	123.11 (12)
C6—C1—C2	117.60 (12)	C12—C11—C10	120.59 (12)
C6—C1—C7	122.88 (12)	C12—C11—H11A	119.7
C2—C1—C7	119.51 (12)	C10—C11—H11A	119.7
C3—C2—C1	121.58 (13)	O3—C12—C11	125.08 (12)
C3—C2—H2A	119.2	O3—C12—C13	114.66 (12)
C1—C2—H2A	119.2	C11—C12—C13	120.25 (11)
C2—C3—C4	119.93 (13)	O4—C13—C14	125.14 (12)
C2—C3—H3A	120.0	O4—C13—C12	115.48 (12)
C4—C3—H3A	120.0	C14—C13—C12	119.37 (13)
O1—C4—C5	122.41 (12)	C13—C14—C15	119.73 (13)
O1—C4—C3	118.02 (12)	C13—C14—H14A	120.1
C5—C4—C3	119.57 (13)	C15—C14—H14A	120.1
C6—C5—C4	120.02 (12)	C10—C15—C14	121.98 (12)
C6—C5—H5A	120.0	C10—C15—H15A	119.0
C4—C5—H5A	120.0	C14—C15—H15A	119.0
C5—C6—C1	121.28 (12)	O3—C16—H16A	109.5
C5—C6—H6A	119.4	O3—C16—H16B	109.5
C1—C6—H6A	119.4	H16A—C16—H16B	109.5
O2—C7—C1	120.15 (12)	O3—C16—H16C	109.5
O2—C7—C8	120.51 (12)	H16A—C16—H16C	109.5
C1—C7—C8	119.34 (11)	H16B—C16—H16C	109.5
C9—C8—C7	121.42 (12)	O4—C17—H17A	109.5
C9—C8—H8A	119.3	O4—C17—H17B	109.5
C7—C8—H8A	119.3	H17A—C17—H17B	109.5
C8—C9—C10	128.19 (12)	O4—C17—H17C	109.5
C8—C9—H9A	115.9	H17A—C17—H17C	109.5
C10—C9—H9A	115.9	H17B—C17—H17C	109.5
			
C6—C1—C2—C3	0.2 (2)	C8—C9—C10—C11	2.9 (2)
C7—C1—C2—C3	179.60 (14)	C15—C10—C11—C12	0.59 (19)
C1—C2—C3—C4	−0.7 (2)	C9—C10—C11—C12	−178.34 (12)
C2—C3—C4—O1	179.88 (14)	C16—O3—C12—C11	4.2 (2)
C2—C3—C4—C5	0.6 (2)	C16—O3—C12—C13	−175.33 (14)
O1—C4—C5—C6	−179.27 (13)	C10—C11—C12—O3	−178.55 (13)
C3—C4—C5—C6	0.0 (2)	C10—C11—C12—C13	1.0 (2)
C4—C5—C6—C1	−0.5 (2)	C17—O4—C13—C14	4.3 (3)
C2—C1—C6—C5	0.3 (2)	C17—O4—C13—C12	−176.96 (16)
C7—C1—C6—C5	−178.98 (13)	O3—C12—C13—O4	−0.94 (19)
C6—C1—C7—O2	172.43 (13)	C11—C12—C13—O4	179.51 (13)
C2—C1—C7—O2	−6.9 (2)	O3—C12—C13—C14	177.85 (13)
C6—C1—C7—C8	−7.6 (2)	C11—C12—C13—C14	−1.7 (2)
C2—C1—C7—C8	173.06 (12)	O4—C13—C14—C15	179.55 (14)
O2—C7—C8—C9	−15.9 (2)	C12—C13—C14—C15	0.9 (2)
C1—C7—C8—C9	164.20 (13)	C11—C10—C15—C14	−1.4 (2)
C7—C8—C9—C10	178.09 (12)	C9—C10—C15—C14	177.55 (13)
C8—C9—C10—C15	−176.03 (14)	C13—C14—C15—C10	0.7 (2)

### Hydrogen-bond geometry (Å, °)

**Table:**

*D*—H···*A*	*D*—H	H···*A*	*D*···*A*	*D*—H···*A*
O1—H1A···O2^i^	0.82	1.90	2.7154 (15)	176
C6—H6A···O4^ii^	0.93	2.46	3.2803 (16)	147

Symmetry codes: (i) *x*−1/2, −*y*+1/2, −*z*+1; (ii) −*x*+1, *y*−1/2, −*z*+1/2.

### Table 2 Selected geometric parmeters (Å): Cg···Cg π stacking interactions, Cg1 is the centroid of ring C1—C6 [Symmetry codes: (i) 1-x, -y, 1-z]

**Table:**

*Cg*I···*Cg*J	Cg···Cg (Å)	CgI Perp (Å)	Cgj Perp (Å)	Slippage (Å)
Cg1···Cg1^i^	3.7386 (9)	-3.3959 (6)	-3.3958 (6)	1.56 (4)
